# Adrenoleukodystrophy Newborn Screening in the Netherlands (SCAN Study): The X-Factor

**DOI:** 10.3389/fcell.2020.00499

**Published:** 2020-06-17

**Authors:** Rinse W. Barendsen, Inge M. E. Dijkstra, Wouter F. Visser, Mariëlle Alders, Jet Bliek, Anita Boelen, Marelle J. Bouva, Saskia N. van der Crabben, Ellen Elsinghorst, Ankie G. M. van Gorp, Annemieke C. Heijboer, Mandy Jansen, Yorrick R. J. Jaspers, Henk van Lenthe, Ingrid Metgod, Christiaan F. Mooij, Elise H. C. van der Sluijs, A. S. Paul van Trotsenburg, Rendelien K. Verschoof-Puite, Frédéric M. Vaz, Hans R. Waterham, Frits A. Wijburg, Marc Engelen, Eugènie Dekkers, Stephan Kemp

**Affiliations:** ^1^Department of Clinical Chemistry, Laboratory Genetic Metabolic Diseases, Amsterdam UMC, Amsterdam Gastroenterology and Metabolism, University of Amsterdam, Amsterdam, Netherlands; ^2^Pediatric Metabolic Diseases, Amsterdam UMC, Emma Children’s Hospital, University of Amsterdam, Amsterdam, Netherlands; ^3^Centre for Health Protection, National Institute for Public Health and the Environment (RIVM), Bilthoven, Netherlands; ^4^Department of Clinical Genetics, Amsterdam UMC, Amsterdam Reproduction & Development, University of Amsterdam, Amsterdam, Netherlands; ^5^Department of Clinical Chemistry, Neonatal Screening Laboratory, Endocrine Laboratory, Amsterdam UMC, Amsterdam Gastroenterology and Metabolism, University of Amsterdam, Amsterdam, Netherlands; ^6^Reference Laboratory for Neonatal Screening, Centre for Health Protection, National Institute for Public Health and the Environment (RIVM), Bilthoven, Netherlands; ^7^Centre for Population Screening, National Institute for Public Health and the Environment (RIVM), Bilthoven, Netherlands; ^8^Department of Clinical Chemistry, Endocrine Laboratory, Amsterdam UMC, Amsterdam Gastroenterology and Metabolism, Vrije Universiteit Amsterdam, Amsterdam, Netherlands; ^9^Department for Vaccine Supply and Prevention Programmes, National Institute for Public Health and the Environment (RIVM), Bilthoven, Netherlands; ^10^Department of Pediatric Endocrinology, Amsterdam UMC, Emma Children’s Hospital, University of Amsterdam, Amsterdam, Netherlands; ^11^Department of Pediatric Neurology, Amsterdam UMC, Amsterdam Leukodystrophy Center, Emma Children’s Hospital, Amsterdam Neuroscience, University of Amsterdam, Amsterdam, Netherlands

**Keywords:** adrenoleukodystrophy, peroxisomes, newborn screening, neonatal, gender, heel prick, dried bloodspots, X chromosome

## Abstract

X-linked adrenoleukodystrophy (ALD) is a devastating metabolic disorder affecting the adrenal glands, brain and spinal cord. Males with ALD are at high risk for developing adrenal insufficiency or progressive cerebral white matter lesions (cerebral ALD) at an early age. If untreated, cerebral ALD is often fatal. Women with ALD are not at risk for adrenal insufficiency or cerebral ALD. Newborn screening for ALD in males enables prospective monitoring and timely therapeutic intervention, thereby preventing irreparable damage and saving lives. The Dutch Ministry of Health adopted the advice of the Dutch Health Council to add a boys-only screen for ALD to the newborn screening panel. The recommendation made by the Dutch Health Council to only screen boys, without gathering any unsolicited findings, posed a challenge. We were invited to set up a prospective pilot study that became known as the SCAN study (SCreening for ALD in the Netherlands). The objectives of the SCAN study are: (1) designing a boys-only screening algorithm that identifies males with ALD and without unsolicited findings; (2) integrating this algorithm into the structure of the Dutch newborn screening program without harming the current newborn screening; (3) assessing the practical and ethical implications of screening only boys for ALD; and (4) setting up a comprehensive follow-up that is both patient- and parent-friendly. We successfully developed and validated a screening algorithm that can be integrated into the Dutch newborn screening program. The core of this algorithm is the “X-counter.” The X-counter determines the number of X chromosomes without assessing the presence of a Y chromosome. The X-counter is integrated as second tier in our 4-tier screening algorithm. Furthermore, we ensured that our screening algorithm does not result in unsolicited findings. Finally, we developed a patient- and parent-friendly, multidisciplinary, centralized follow-up protocol. Our boys-only ALD screening algorithm offers a solution for countries that encounter similar ethical considerations, for ALD as well as for other X-linked diseases. For ALD, this alternative boys-only screening algorithm may result in a more rapid inclusion of ALD in newborn screening programs worldwide.

## Introduction

X-linked adrenoleukodystrophy (ALD) is a severe inborn error of metabolism caused by a mutation in the *ABCD1* gene located on the X chromosome (Mosser et al., 1993). The *ABCD1* gene codes for the peroxisomal transmembrane protein (ABCD1 protein) that transports very long-chain fatty acids (VLCFA, ≥C22:0) into the peroxisome. A defect in *ABCD1* results in impaired VLCFA beta-oxidation and consequently an accumulation of VLCFA in plasma and tissues (Singh et al., 1984; Moser et al., 1998; Huffnagel et al., 2017). ALD can affect the brain, adrenal glands and the spinal cord (Kemp et al., 2016). ALD has an estimated prevalence of 1 in 15.000–17.000 births (Bezman and Moser, 1998; Moser et al., 2016). About 35% of boys with ALD develop progressive cerebral white matter lesions (cerebral ALD) between the 3rd and 10th year of life, and it is well documented that this type of brain pathology frequently occurs in adulthood, as well (Moser et al., 2000; van Geel et al., 2001; de Beer et al., 2014; Huffnagel et al., 2019b). If left untreated, cerebral ALD is often fatal (Moser et al., 2007). Hematopoietic stem cell transplantation (HSCT) can stop the progression of cerebral ALD, provided the procedure is performed at an early stage of the disease (Aubourg et al., 1990; Raymond et al., 2019). Unfortunately, this therapeutic window is narrow and often missed because of delayed diagnosis (Polgreen et al., 2011; Engelen et al., 2012).

For male ALD patients, the lifetime prevalence of adrenal insufficiency is 80–90%; with 50% between the age of 5 months to 10 years (Dubey et al., 2005; Huffnagel et al., 2019b). Failure to recognize adrenal insufficiency at an early stage can lead to severe clinical symptoms. Adrenal insufficiency can be treated relatively easily with oral hormone replacement (Regelmann et al., 2018). In contrast to males, women with ALD have a very low risk (<1%) for developing adrenal insufficiency or cerebral ALD (Moser et al., 2001). Newborn screening for ALD allows prospective monitoring and timely therapeutic intervention, thus preventing irreversible damage and saving lives.

Following the passage of Aidan’s Law in the Spring of 2013, New York was the first United States state to include ALD in its newborn screening (NBS) panel (Moser et al., 2016). ALD NBS in New York State is accomplished via a three-tier algorithm (Vogel et al., 2015). Tier 1 consists of a high-throughput flow injection analysis tandem mass spectrometry (FIA–MS/MS) of C26:0 lysophosphatidylcholine (C26:0-LPC) (Hubbard et al., 2009). Screen positive samples are then reanalyzed in tier 2, which consists of a highly specific high-performance liquid chromatography–tandem mass spectrometry (HPLC-MS/MS) (Turgeon et al., 2015). If C26:0-LPC is also detected as elevated in the second tier, samples continue to tier 3, which is sequencing of the *ABCD1* gene (Boehm et al., 1999). In 2016, ALD was added to the United States Recommended Uniform Screening Panel (RUSP) (Moser et al., 2016). As of March 2020, 16 states in the United States have started newborn screening for ALD.

**FIGURE 1:**
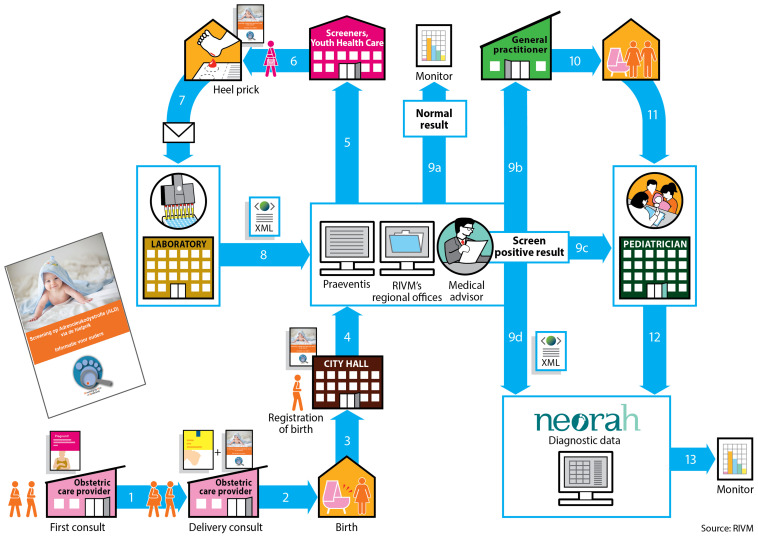
The organization of the Dutch newborn screening process. The obstetric care provider is responsible for informing the pregnant women about the newborn screening. In the third trimester the obstetric care provider informs the parents about the newborn screening and the SCAN study and provides them with the information folders. After the baby is born, its birth is registered at City Hall. At City Hall, the information folder about the newborn screening and the information folder about the SCAN study will be given to the parents. After registration the local Youth Health Care receives notification to perform the heel prick when the newborn is 3 to 7 days old. The heel prick for the newborn screening is performed by Youth Health Care, a midwife or a maternity nurse if the child is at home. A nurse will perform the heel prick if the child is in the hospital. Before performing the heel prick, the informed consent request for the newborn screening and the ALD-screening are handed over separately. If necessary, the SCAN study folder can be provided again. For the Dutch newborn screening system, the Netherlands is divided into 5 regions. Each region has its own screening laboratory (Figure 2). At these screening laboratories all incoming dried blood spots cards are processed, registered and analyzed. The results are entered into the national registration system (Praeventis) of the RIVM. When the screening result is abnormal, the region’s medical advisor is informed and the advisor assesses the laboratory results. The medical advisor will contact both a (specialized) pediatrician as well as the newborn’s general practitioner (GP). The child is referred to a (specialized) pediatrician by the GP. The medical advisor registers the positive screening result in the national NEOnatal Registration of Abnormalities found in Heel prick screening (NEORAH).

The NBS program in the Netherlands first started in 1974 with the screening of phenylketonuria (PKU) and currently includes 22 conditions (Table 1). The Dutch NBS program is coordinated by the Center for Population Screening (RIVM-CvB). In the Netherlands newborn screening is voluntary and based upon informed consent provided by the newborn’s parents. Participation in the newborn screening program is high: in 2018, 99.1% of all newborns (168.565) were screened (Van der Ploeg et al., 2019). The Dutch newborn screening system is a complex enterprise, with many involved parties and associated steps (Figure 1). Newborn screening and pediatric healthcare (<18 years of age) in the Netherlands are free of costs for patients and parents.

**TABLE 1:** Conditions included in the Dutch newborn screening program.

Year of introduction	Disease
1974	Phenylketonuria (PKU)
1981	Congenital hypothyroidism (CH)
2002	Congenital adrenal hyperplasia (CAH)
2007	Sickle cell Disease (SCD)
2007	Biotinidase deficiency (BIO)
2007	Galactosemia (GAL)
2007	Glutaric acidemia type 1 (GA-1)
2007	HMG CoA lyase deficiency (HMG)
2007	Isovaleric acidemia (IVA)
2007	Long-chain hydroxyacyl-CoA dehydrogenase deficiency (LCHAD)
2007	Maple syrup urine disease (MSUD)
2007	Medium-chain acyl-CoA dehydrogenase deficiency (MCADD)
2007	3-methylcrotonyl-CoA carboxylase deficiency (3-MCC)
2007	Malonyl-CoA decarboxylase deficiency (MCD)
2007	Tyrosinemia Type 1
2007	Very long-chain acyl-CoA dehydrogenase deficiency (VLCADD)
2011	Cystic Fibrosis (CF)
2017	Hemoglobin H disease
2017	Beta-thalassemia major
2019	Carnitine palmitoyltransferase I deficiency (CPT1)
2019	Methylmalonic acidemia (MMA)
2019	Propionic acidemia (PA)

In 2015, the Dutch Ministry of Health adopted the advice of the Dutch Health Council to add 14 new conditions to the newborn screening panel, including ALD (Health Council of the Netherlands, 2015). For ALD, the Dutch Health Council advised to only screen boys. They stated in their report: “Screening for X-ALD is useful only in male newborns, as symptoms in women usually develop later and are untreatable (..). The possibility to screen only male newborns without loss of efficiency should be studied” (Health Council of the Netherlands, 2015).

Gender-specific screening has, to our knowledge, not been implemented before in any NBS program worldwide. The ALD-group at Amsterdam UMC was invited to set up a prospective pilot study to develop a screening algorithm that successfully identifies males with ALD, while at the same time ensuring that unsolicited findings were not identified. Furthermore, these “guardrails” should not adversely impact the overall efficiency of the NBS program. The acronym of the prospective pilot study became the SCAN study (SCreening for ALD in the Netherlands). The SCAN study is a collaboration between the ALD-group at Amsterdam UMC and the National Institute for Public Health and the Environment (RIVM).

**FIGURE 2:**
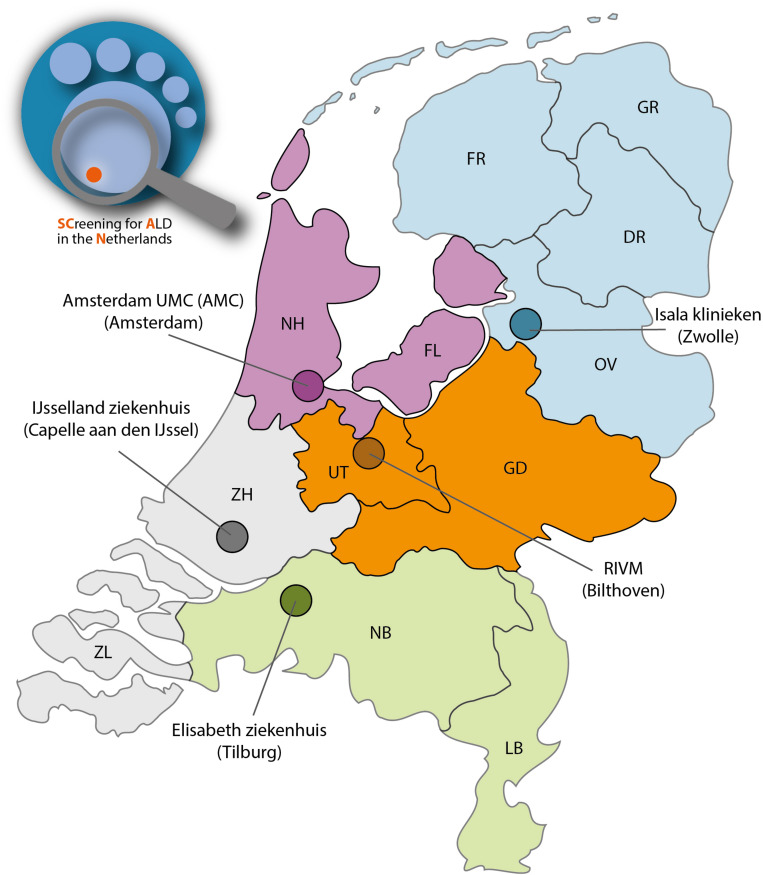
The SCAN study will be performed in 2 NBS screening regions consisting of 4 provinces: Noord-Holland (NH), Flevoland (FL), Utrecht (UT) and Gelderland (GD). Two newborn screenings laboratories are involved: Amsterdam UMC (location AMC) and Bilthoven (RIVM).

The 14 new conditions, including ALD, will be added to the Dutch NBS program in a phased manner, with 1–3 diseases per year (Health Council of the Netherlands, 2015). For the Dutch newborn screening system, the Netherlands is divided into 5 regions. Each region has its own screening laboratory (Figure 2). During the pilot study an estimated 70.000 newborns will be screened in two screenings laboratories (SL): Amsterdam (Amsterdam UMC) and Bilthoven (RIVM). The specific objectives for the SCAN study are: (1) designing a boys-only screening algorithm that identifies males with ALD and without unsolicited findings; (2) implementing this algorithm in the structure of the Dutch NBS program without adversely affecting the performance of the current NBS; (3) assessing the practical and ethical implications of screening only boys for ALD; and (4) setting up a comprehensive follow-up that is both patient- and parent-friendly.

To organize the SCAN study a multidisciplinary project group was formed. This group consists of a project leader, a pediatric neurologist specialized in leukodystrophies, the NBS program manager, the NBS account manager, a medical ethicist, a clinical geneticist, a pediatric endocrinologist, a medical advisor of the NBS program, the heads of the two participating newborn screening laboratories, communications specialists and a PhD-student with a medical background.

## Materials and Methods

### Samples

Anonymized dried blood spots (DBS) cards from 250 control newborns were provided by the Dutch neonatal screening program with permission of the relevant ethical committee (WONHS-2019-4). ALD DBS cards were prepared from anti-coagulated (EDTA) whole venous blood specimens obtained from 58 male ALD patients currently participating in a prospective natural history study (referred to as “The Dutch ALD cohort”; IRB: METC 2018_310). Written informed consent was received from each patient. DBS cards were prepared within 24 h after venipuncture, as described previously (Singh et al., 1984). For the adult control group, we collected all routine C26:0-LPC measurements performed at the Laboratory Genetic Metabolic Diseases in the Academic Medical Center. Measurements of C26:0-LPC in DBS of patients diagnosed with a peroxisomal disorder were excluded. The remainder of the C26:0-LPC measurements were combined and labeled as the control group (126 samples). Approval of the Institutional Review Board for the analysis of C26:0-LPC in DBS cards was not required, since all measurements were performed as part of diagnostic procedures or standard patient care and data were anonymized for the purpose of further analysis.

### Analysis of C26:0-LPC by Flow Injection Analysis-Tandem Mass Spectrometry (FIA-MS/MS)

The analysis of C26:0-LPC is performed using the Neobase 2 newborn screening kit (PerkinElmer) following the manufacturer’s instructions. Single 3.2 mm discs are punched from DBS and transferred into 96-well plates. 125 μL of the PerkinElmer Neobase 2 extraction working solution (EWS) is added to each well. The microplate is covered with an adhesive microplate cover and shaken for 30 min with 650–750 rpm at 45°C. The microplate cover is removed and 100 μL is transferred to a new microplate and covered with an adhesive microplate cover before being analyzed with a Waters Xevo TQD.

### DNA-Isolation for DBS

In a 1.5 mL Eppendorf tube, a single disc of a 3.2 mm dried bloodspot is extracted with 280 μL ATL buffer and 20 μL proteinase K (QIAamp DNA Investigator Kit, Qiagen). The tube is vortex mixed and incubated in a heating block at 56°C for 1 h while shaking at 900rpm. Then the tube is briefly centrifuged and the liquid phase is transferred to a new 2 mL safe-lock Eppendorf tube. After the lysis procedure, DNA isolation is performed using the QIAcube according to the manufacturer’s instructions.

### Determining the Number of X-Chromosomes

For determining the number of X-chromosomes present in tier 1 screen positive DBS, a variety of commercial kits are available. We decided to use the Devyser Resolution XY v2 kit (Devyser, Stockholm, Sweden) because this test determines the ratio of 3 different non-polymorphic markers that are present both on the X chromosome and an autosomal chromosome. Three ratios are determined: X to 7 (T1), 2 to X (T2) and X to 3 (T3). The ratio of T2 is inverted compared to T1 and T3, which is due to the length of the amplicons and the subsequent visualization. A ratio 1:1 indicates a female and a ratio 1:2 (or 2:1) indicates a male sample. The kit, however, is also used to screen for other chromosomal disorders, which are considered unsolicited findings in the Dutch screening program. As a solution, Devyser made modifications to the analysis settings to shield all other markers. The PCR is performed following the manufacturer’s instructions. After the PCR reaction, the sample is prepared for an ABI3500 Genetic Analyzer (Thermo Fisher Inc). 1,5 μL of the PCR-product is transferred to a new PCR reaction tube and 15 μL activated reaction mix is added. The activated reaction is prepared fresh by mixing 3 μL 560 Sizer Orange and 100 μL HiDi^TM^ Formamide. The sample is vortex mixed, briefly centrifuged and analyzed on the ABI3500 for 25 min at 60°C, injection voltage 1.6 kV, injection time 15 s and run voltage 19.5 kV. Results are analyzed using Genemapper 5 software (Thermo Fisher Scientific Inc) using the kit manufacturer’s adjusted software settings. In case one marker fails and the two remaining markers are in agreement, the test is considered successful.

### Analysis of C26:0-LPC by High-Performance Liquid Chromatography-Tandem Mass Spectrometry (HPLC-MS/MS)

C26:0-LPC levels are analyzed as described earlier by Huffnagel et al. with minor modifications to facilitate analysis of material obtained from a 3.2 mm disc (Huffnagel et al., 2017).

### *ABCD1* Gene-Sequencing

*ABCD1* gene mutation analysis is performed according the protocol described by Boehm et al. (1999).

## Results and Discussion

### Informed Consent/Information/Education

Participation in the SCAN study is based on informed consent. Therefore, we developed an information folder for parents which we distributed to all participating obstetric care providers, hospitals, city halls and screeners from youth health care. In the participating regions, the folder was inserted in the existing information folder about the newborn screening program and was distributed to the relevant healthcare providers. In addition, we included a cover letter for the professionals containing additional information and tips on how to communicate this information to the parents. Furthermore, a website was developed www.scanstudie.nl. Using these resources, we enable parents to provide or deny informed consent for participating in the SCAN study.

To inform healthcare professionals working in the NBS program about the SCAN study we organized two events. During these information-dissemination events, healthcare professionals were given background information about ALD, including the rationale underlying the decision made by the Health Council to only screen boys with time for Q&A.

### Screening Algorithm

Newborn screening for ALD is based on the quantification of C26:0-LPC in newborn dried bloodspots (Hubbard et al., 2006, 2009; Huffnagel et al., 2017). The Dutch ALD-screening is inspired by the 3-tier screening algorithm developed by New York State (Vogel et al., 2015). In New York State, the first tier consists of quantification of C26:0-LPC by FIA-MS/MS (Vogel et al., 2015; Huffnagel et al., 2017). For determining the cut-off level for tier 1 we evaluated the normal distribution of C26:0-LPC measured in Dutch newborns and combined this with the tier 1 cut-off level of New York State (provided by dr. Joseph Orsini via personal communications). However, since we also have to adequately test the logistics of the entire screening process and the remaining tiers, we ensured that a sufficient number of samples will reach these tiers by lowering the cut-off level slightly. This results in higher numbers of DBS screen positives in tier 1 and thereby more samples to run through the next tiers. If C26:0-LPC is above the cut-off level, the sample continues to tier 2. In tier 2, C26:0-LPC is measured using the more specific HPLC-MS/MS (Hubbard et al., 2006, 2009; Turgeon et al., 2015; Vogel et al., 2015). Tier 3 consists of *ABCD1*-gene sequencing (Boehm et al., 1999). If no mutation in the *ABCD1* gene is found, additional diagnostic tests will be initiated toward a diagnosis causing the elevated VLCFA. Since one of the objectives of the SCAN study was the development of an algorithm that enables the screening of only boys for ALD, we adjusted the New York State algorithm.

### Sex Determination

Sex determination is a complex screening algorithm challenge. An estimated 0.018% of all newborns is born with a condition in which chromosomal sex is inconsistent with phenotypic sex, or in which the phenotype is not classifiable as either male or female (Sax, 2002). With an average annual birth rate of 175.000 newborns in the Netherlands, the gender of an estimated 32 newborns per year may be wrongly classified. Furthermore, errors occur during the administrative procedure: sometimes the gender on the heel prick cards is filled out incompletely, or filled out incorrectly or the answer itself is unclear (e.g., due to the blood stains obscuring the text). In addition, errors can occur during any of the administrative registrations of the newborn. We estimated the rate of error to be up to 3–4%. Therefore, a new highly reliable test was needed that would allow us to distinguish boys from girls genetically, without importing unsolicited findings. We considered a test based on assessing the presence of a Y chromosome, however, that test does not distinguish 46,XY boys from newborns with Klinefelter syndrome (47,XXY) which would be an unsolicited finding. Therefore, we decided to determine the number of X chromosomes. Of the various commercial kits that are available we decided to use the Resolution XY v2 kit from Devyser. The tier 2 test is referred to as the “X-counter.” A bloodspot from a girl with Turner syndrome (45,X) would pass the test and continue to tier 3. However, when C26:0-LPC levels are normal in tier 3, this newborn would not continue in the screen and will therefore not be diagnosed with Turner syndrome in the absence of ALD. It is expected that a girl with Turner syndrome and ALD will have the same risk of developing cerebral ALD as boys with ALD. Therefore, she should receive the same follow-up. In addition, the *a priori* chance of a girl having Turner syndrome combined with ALD is extremely small.

**FIGURE 3:**
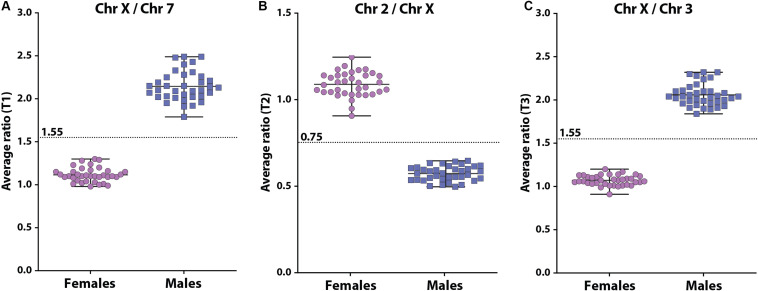
Scatterplots of the 3 ratios between the 3 autosomes and the X chromosome [**(A)**: X to 7, **(B)**: 2 to X, **(C)**: X to 3] determined in DNA isolated from DBS in females (purple circles) and males (blue squares). The cut-offs for each test are indicated by the dashed line.

The X-counter determines the ratio between 3 different autosomes (chromosome 2, 3, and 7, respectively) and the X chromosome using 3 different non-polymorphic markers that are present on both the X chromosome and an autosomal chromosome (Figure 3). To validate the X-counter and determine the cut-off values for the 3 markers, we determined the 3 ratios in 72 DBS with known gender (36 males and 36 females). In females, the mean ratio’s for X to 7 (T1), 2 to X (T2) and X to 3 (T3) were 1.12, 1.09, and 1.07, respectively (Figure 3). In males, the mean ratio’s for X to 7 (T1), 2 to X (T2) and X to 3 (T3) were 2.15, 0.57, and 2.06, respectively (Figure 3). The ratio of T2 is inverted compared to T1 and T3, which is due to the length of the amplicons and the subsequent visualization. Based on these results the cut-off for T1 and T3 were set at 1.55 and for T2 at 0.75. Females are defined by T1 < 1.55, T2 > 0.75, and T3 < 1.55. Males are defined by T1 ≥ 1.55, T2 ≤ 0.75, and T3 ≥ 1.55. To prevent the identification of unsolicited findings, such as a micro deletion that causes one of the PCR amplifications to fail, we decided that in case there are inconsistencies in the results of the three markers, the test outcome will be based on the two markers that show the same result. Also, in case one marker fails and the two remaining markers are in agreement, the test is considered successful. The Devyser XYv2 kit is a diagnostic product that is also used for determination of aneuploidies in X and Y chromosomes. In the SCAN study these would be considered as unsolicited findings. Therefore, settings in the analysis software were modified by Devyser to ensure that only the ratio of the three autosomes and the X chromosome are calculated. Finally, to verify whether the test generates an unambiguous result in a newborn DBS, we analyzed 72 anonymized newborn DBS of which the gender was unknown to us. All samples could be labeled either XY or XX. In 69 analyses, all 3 tests were informative. In 3 analyses, T2 failed, but T1 and T3 were unambiguous in their outcome. In the real-life screening, the results of the X-counter are entered into Praeventis, the national registration system of the RIVM. Output of the test to Praeventis is 1 X chromosome or >1 X chromosomes. Samples with 1 X chromosome continue to tier 3.

### HPLC-MS/MS Analysis of C26:0-LPC

**FIGURE 4:**
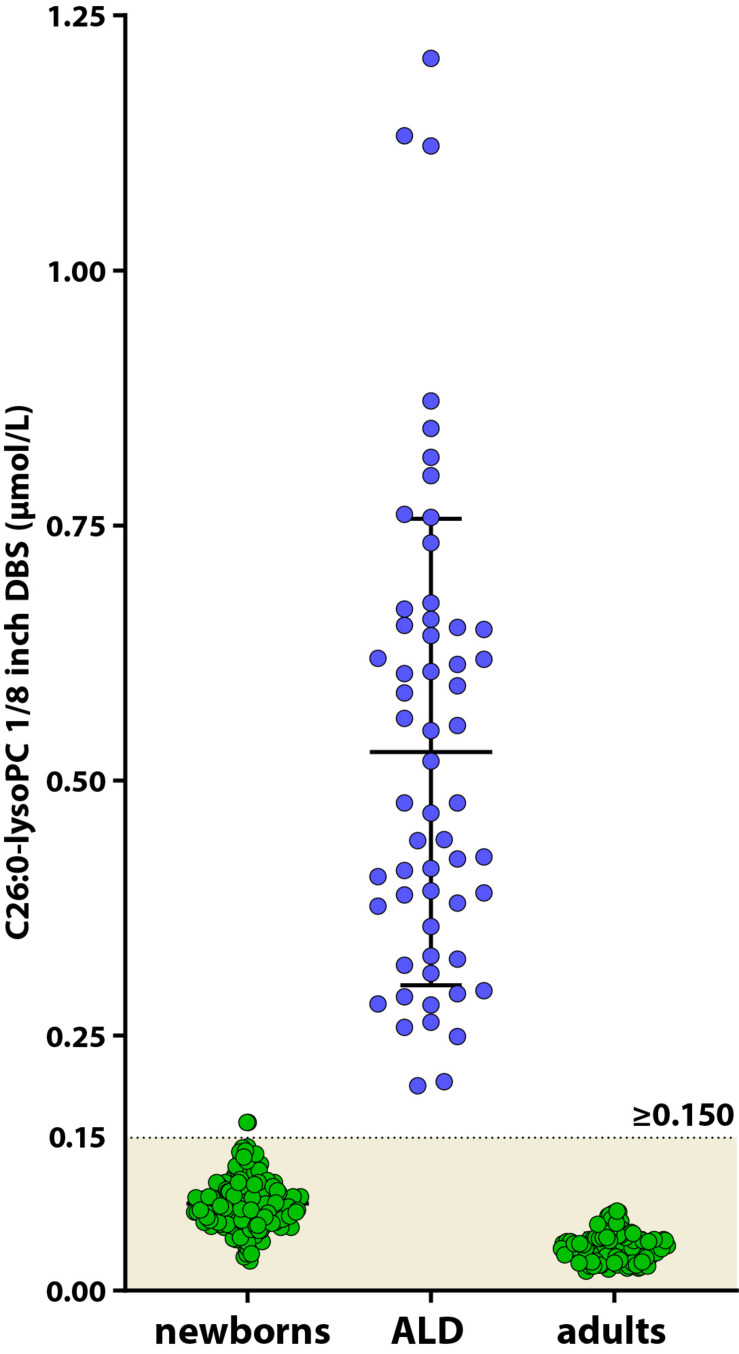
Scatterplot of C26:0-LPC levels in DBS from control newborns (green circles, left), ALD patients (blue circles) and adult controls (green circles, right) determined by HPLC-MS/MS. The control range for C26:0-LPC in newborns is indicated by the colored area.

Due to the presence of an unknown isobaric interferent, the majority of the C26:0-LPC measurements with FIA-MS/MS are falsely elevated (Hubbard et al., 2009; Turgeon et al., 2015). Therefore, all newborns with a screen positive tier 1 and one X chromosome are also screened with the more specific and sensitive HPLC-MS/MS analysis of C26:0-LPC. To establish reference values for newborn controls and to determine the cut-off value for C26:0-LPC in newborns, 250 anonymized DBS from newborns and 58 ALD patients from the Dutch ALD-cohort were tested for C26:0-LPC by HPLC-MS/MS. Ideally, we would have used DBS from newborns with ALD. However, these were not available. The mean level in newborn controls was 0.086 μmol/L (0.029–0.165 μmol/L) and the mean level in ALD patients was 0.527 μmol/L (range 0.201–1.208 μmol/L). In addition, C26:0-LPC levels were determined in 126 adult controls. In adult controls C26:0-LPC levels were 0.042 μmol/L (range 0.019–0.078 μmol/L), which is lower when compared to newborn controls. In blood, C26:0-LPC is present predominantly in the membranes of red blood cells (Nishio et al., 1986; Tanaka et al., 1989). Newborns have a higher red blood cell count than adults (Cheng et al., 2004). Therefore, the lower level of C26:0-LPC in adults likely correlates with the decrease in red blood cells as the child matures. Based on these results we define samples C26:0-LPC ≥ 0.150 μmol/L as screen positive (Figure 4). During the pilot screening we expect to find higher C26:0-LPC levels in DBS of newborns with ALD than our samples from adults with ALD due to the decline of C26:0-LPC over age. Therefore, we may have more samples going to tier 4 than expected. If this occurs, the cut-off will be adjusted. One sample from the newborn control group had an out of range C26:0-LPC level. We could not investigate the potential underlying cause for this, because we only had a single anonymized DBS available. Newborns with a C26:0-LPC above 0.150 μmol/L will continue to the *ABCD1* gene sequencing tier 4.

### Mutation Analysis

Elevated C26:0-LPC is indicative for a defect in peroxisomal beta-oxidation. However, elevated C26:0-LPC levels are not specific for ALD. C26:0-LPC is also elevated in patients with Zellweger Spectrum Disorder, ACOX1 or HSD1B4 deficiency, CADDS, ACBD5 deficiency and Aicardi Goutières Syndrome (Armangue et al., 2017; Ferdinandusse et al., 2017; Klouwer et al., 2017). Therefore, to diagnose boys with ALD only, an extra test is required: sequencing of each of the 10 exons of the *ABCD1* gene. If this results in the identification of either a pathogenic mutation in the *ABCD1* gene or a variant of unknown significance (VUS) in the *ABCD1* gene, the ALD screening will be classified as abnormal and the newborn will be referred to the Pediatric Neurology Department of the Amsterdam UMC, location AMC.

### Ethics

In male ALD patients, pathogenic *ABCD1* mutations have no prognostic value with respect to the clinical outcome of an individual (Kemp et al., 2016). Boys may benefit significantly from early diagnosis, since this enables close monitoring for adrenal insufficiency and cerebral ALD, allowing timely initiation of lifesaving treatment. For women with ALD the lifetime prevalence of myelopathy is 80–90% (Engelen et al., 2012). In general, the age of onset is between the age of 40–60 years (Engelen et al., 2014; Habekost et al., 2014). Currently there is no curative treatment for myelopathy. Less than 1% of women with ALD will develop cerebral ALD or adrenal insufficiency (Engelen et al., 2012).

The Dutch Health Council based its advice on the international criteria for population screening by Wilson and Jungner and the additional criteria established by the WHO (Wilson et al., 1968; Andermann et al., 2008). This advice has resulted in a different ALD newborn screening algorithm when compared to the United States, since in the United States both boys and girls are screened for ALD. There are different ethical views related to this advice, both from a population screening perspective and from an ALD perspective. It is important to make a thorough assessment of all ethical considerations. For that purpose, we shall explore this in a separate study within the scope of the SCAN study.

### Dutch ALD Newborn Screening Algorithm

**FIGURE 5:**
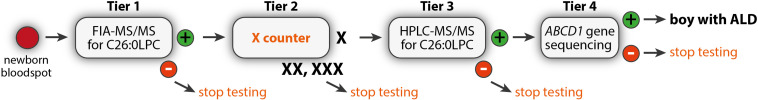
The 4-tier Dutch ALD newborn screening algorithm.

Because of the presence of a yet unknown isobaric interferent when measuring C26:0-LPC by FIA-MS/MS, the majority of screen positive samples by FIA-MS/MS will be false positive (Turgeon et al., 2015). Therefore, screen positive samples are subsequently reanalyzed by HPLC-MS/MS. HPLC-MS/MS separates the isobaric interferent from C26:0-LPC. The high specificity but low sensitivity of tier 1 results in girls not yet effectively being screened for C26:0-LPC in tier 1. As a consequence, the boys-only criterion from the Dutch Health Council is still met after FIA-MS/MS. Because HPLC-MS/MS is a very sensitive test for identifying elevated C26:0-LPC, girls must be excluded prior to this test. Based on these criteria and conditions, we positioned the X-counter as tier 2 component in the screening algorithm (Figure 5). Tier 1 is a multiplex tier that is also used in the screening for other metabolic diseases. Therefore, placing the X-counter after tier 1 ensures that the tier 1 test is not delayed. Hence, the algorithm fulfills the requirement that the efficacy of existing NBS programs for other conditions may not be adversely impacted. Tier 4 consists of *ABCD1* gene sequencing. Only once a pathogenic variant, or a variant of unknown significance is identified, the newborn is referred to the Amsterdam UMC for follow-up.

During the pilot, tier 1 will be conducted only in the newborn screening laboratories of Amsterdam and Bilthoven. Screen positive samples are sent to the laboratory of Clinical Genetics at the Amsterdam UMC where tier 2 is performed. Finally, tier 3 and 4 are done at the laboratory Genetic Metabolic Diseases, at the Amsterdam UMC.

In the Netherlands, the newborn screening process (i.e., the time between performing the heel prick until completion of all the tiers and reporting the final results) has to be completed within 5 weeks. To adhere to this national requirement, we developed a strict time schedule.

### Referral

After a positive ALD-screening, the medical advisor informs one of the pediatric neurologists, specialized in leukodystrophies from the Amsterdam Leukodystrophy Center at the Amsterdam UMC by phone. Within 5 working days the newborn and parents are scheduled for a consultation at the outpatient clinic for further diagnostic testing. One working day prior to the scheduled consult, the medical advisor instructs the general practitioner (GP) to inform parents in person and to refer the newborn to the pediatric neurologist. Before the GP visits the family, the GP receives additional information about ALD. Prior to and during the visit of the GP to the parents, the pediatric neurologist serves as back-up for addressing any pressing questions and for providing necessary advice. Since there is no need for immediate medical intervention in newborns with ALD, we focused on providing adequate and patient friendly communication. It is well known parents with anxiety or stress regarding their child’s health resort to dr. Google in times of limited information (Stukus, 2019). Furthermore, browsing the internet in search for information without guidance from a professional mostly increases the levels of stress and anxiety in parents (Stukus, 2019). Therefore, we will not inform parents on a Friday and outpatient clinic appointments will only be scheduled between Tuesday and Friday. This way parents will not be notified on a Friday after which they have to wait an entire weekend for adequate medical information.

### Follow-Up

**FIGURE 6:**
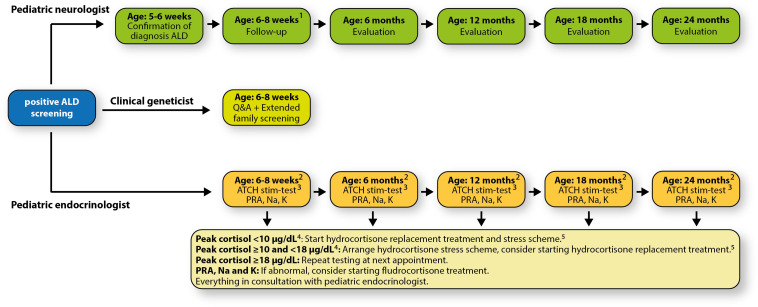
Our patient- and parent-friendly, multidisciplinary, centralized follow-up protocol. ^1^All follow-up appointments will be scheduled on the same day (Wednesday); ^2^Before 10:00 AM; ^3^15 μg/kg/dose, max. 125 μg/dose; ^4^10 μg/dL = 276 nmol/L, 18 μg/dL = 497 nmol/L; ^5^10 mg/m^2^/day in three equal doses (3 times per day 33.3% of the total daily dose); when older than 6 months: 50% early in the morning, and 25% early in the afternoon and evening. Adrenal surveillance protocol adapted and modified from Regelmann et al. (2018). Abbreviations: ACTH, adrenocorticotropic hormone; K, potassium; Na, sodium; PRA, plasma renin activity; Q&A, questions & answers.

We developed a patient- and parent-friendly, multidisciplinary, centralized follow-up protocol. This protocol includes periodic visits to a pediatric neurologist who specializes in leukodystrophies, and a pediatric endocrinologist. Within the structure of this follow-up, a clinical geneticist will also be consulted. Appointments with the pediatric endocrinologist and the clinical geneticist are combined (Figure 6).

The pediatric neurologist coordinates the medical care. After the ALD diagnosis is confirmed, the pediatric neurologist refers the newborn and the parents to the clinical geneticist and the pediatric endocrinologist. The child is seen periodically and evaluated. At the age of 2 years the first cerebral MRI will be performed. Neurological follow-up is based upon recommendations made by Engelen et al. (2012).

Endocrine function follow-up is based upon the recommendations of Regelmann et al. (2018). There are known cases of newborns having signs of adrenal insufficiency already in the first months of life (Eng and Regelmann, 2019). Therefore, the initial intake and evaluation will be scheduled approximately 2 weeks after confirmed diagnosis.

The clinical geneticist will offer extended screening to the newborn’s family and counsel the family, especially the mothers who have an increased risk of being a heterozygous carrier and will develop symptoms later in life (Engelen et al., 2012; Huffnagel et al., 2019a). Moreover, the family will be informed about family planning including prenatal diagnostics and preimplantation diagnostics (PGD). In addition, layperson-targeted education for better understanding ALD inheritance patterns, possibilities, and manifestation is offered.

### Confirming the Diagnosis

**FIGURE 7:**
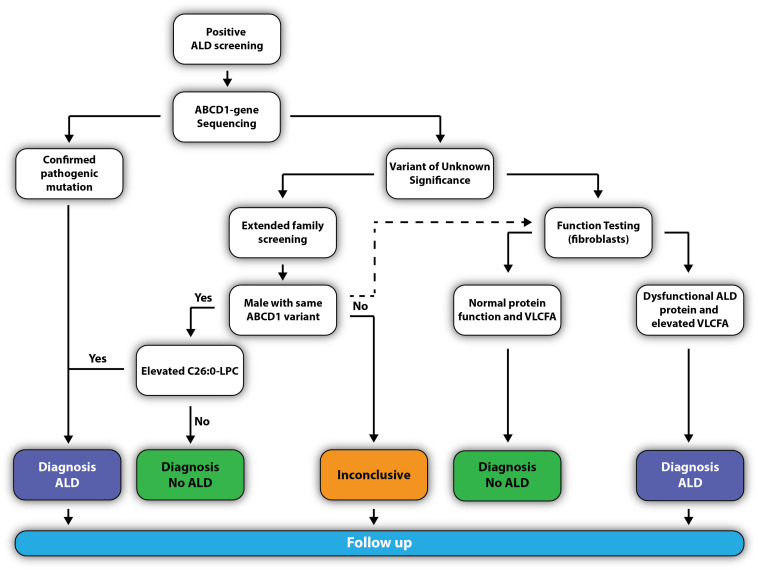
Flowchart to confirm the diagnosis ALD.

The pediatric neurologist will confirm the diagnosis by requesting a confirmatory analysis of C26:0-LPC and *ABCD1* mutation analysis (Figure 7). If the newborn has a known pathogenic missense mutation (previously reported in the ALD mutation database at www.adrenoleukodystrophy.info), or the DNA change results in a clearly deleterious mutation (nonsense, frameshift, deletion), the diagnosis ALD is considered as confirmed.

However, elevated C26:0-LPC is not specific for ALD. Therefore, if a variant of unknown significance (VUS) is found, the newborn should be clinically and metabolically evaluated for symptoms or biomarkers that could point to other peroxisomal storage disorders. Additionally, two scenarios may be followed that can help to elucidate whether the VUS is benign or pathogenic:

(1) Extended family screening to identify family members with the same variant who may be free of clinical signs and symptoms. Because all males with ALD have elevated VLCFA, the identification of a male family member with the same variant and elevated C26:0-LPC will confirm the pathogenic nature of the variant. If the same variant is found in a male relative, but C26:0-LPC levels are normal, the variant is a benign polymorphism and it may be accurately concluded that the newborn does not have ALD. In case a female with the same variant is identified, it is important to realize that 15% of women with ALD have normal plasma (Moser et al., 1999; Engelen et al., 2014). The analysis of C26:0-LPC increases the sensitivity from 85% with the analysis of plasma VLCFA to >99% with the analysis of C26:0-LPC (Huffnagel et al., 2017).
(2) Functional studies on fibroblasts can also help elucidate pathogenic nature of the VUS. To this end, a skin biopsy will be taken from the newborn and fibroblasts will be generated. Functional studies can include visualizing the ALD protein by immunofluorescence (Watkins et al., 1995) and/or Western blotting (Kemp et al., 1996), C26:0-LPC analysis (Huffnagel et al., 2017), VLCFA analysis and/or VLCFA homeostasis by stable isotope-labeled D_3_-C22:0 (van de Beek et al., 2017).

## Conclusion

The recommendations made by the Dutch Health Council to limit ALD screening to males and to avoid diagnosing other disorders characterized by increased VLCFA, posed a challenge. DNA-based gender-specific screening has, to our knowledge, not been implemented before in any NBS program worldwide. We successfully developed a boys-only screening algorithm that identifies males with ALD without unsolicited findings. With respect to newborn screening, different countries may have different ethical views. Therefore, the Dutch boys-only ALD screening algorithm offers a solution for countries that encounter similar ethical considerations, for ALD as well as for other X-linked diseases. For ALD, this alternative boys-only screening algorithm may result in a more rapid inclusion of ALD in newborn screening programs worldwide.

## Data Availability Statement

The raw data supporting the conclusions of this article will be made available by the authors, without undue reservation, to any qualified researcher.

## Ethics Statement

Approval of the Institutional Review Board for the analysis of C26:0-LPC in6 DBS cards was not required, since all measurements were performed as part of diagnostic procedures or standard patient care and data were anonymized for the purpose of further analysis. Written informed consent was received from each patient.

## Author Contributions

ME and SK conceived the project. RB, ME, ED, and SK wrote the manuscript. RB, ID, WV, MA, JB, SC, AG, and SK designed, set up, and performed validation of the X-counter. RB, ID, YJ, HL, ES, FV, ME, and SK performed validation of C26:0-LPC by HPLC-MS/MS. ID, HW, and SK performed validation of ABCD1 sequencing from DBS. RB, WV, MA, JB, AB, MB, EE, AH, MJ, IM, CM, AT, RV-P, FW, ME, ED, and SK organized the SCAN study, referral, and follow-up.

## Conflict of Interest

The authors declare that the research was conducted in the absence of any commercial or financial relationships that could be construed as a potential conflict of interest.
