# Corrigendum: Adrenoleukodystrophy Newborn Screening in the Netherlands (SCAN Study): The X-Factor

**DOI:** 10.3389/fcell.2021.631655

**Published:** 2021-01-28

**Authors:** Rinse W. Barendsen, Inge M. E. Dijkstra, Wouter F. Visser, Mariëlle Alders, Jet Bliek, Anita Boelen, Marelle J. Bouva, Saskia N. van der Crabben, Ellen Elsinghorst, Ankie G. M. van Gorp, Annemieke C. Heijboer, Mandy Jansen, Yorrick R. J. Jaspers, Henk van Lenthe, Ingrid Metgod, Christiaan F. Mooij, Elise H. C. van der Sluijs, A. S. Paul van Trotsenburg, Rendelien K. Verschoof-Puite, Frédéric M. Vaz, Hans R. Waterham, Frits A. Wijburg, Marc Engelen, Eugènie Dekkers, Stephan Kemp

**Affiliations:** ^1^Department of Clinical Chemistry, Laboratory Genetic Metabolic Diseases, Amsterdam UMC, Amsterdam Gastroenterology and Metabolism, University of Amsterdam, Amsterdam, Netherlands; ^2^Pediatric Metabolic Diseases, Amsterdam UMC, Emma Children's Hospital, University of Amsterdam, Amsterdam, Netherlands; ^3^Centre for Health Protection, National Institute for Public Health and the Environment (RIVM), Bilthoven, Netherlands; ^4^Department of Clinical Genetics, Amsterdam UMC, Amsterdam Reproduction & Development, University of Amsterdam, Amsterdam, Netherlands; ^5^Department of Clinical Chemistry, Neonatal Screening Laboratory, Endocrine Laboratory, Amsterdam UMC, Amsterdam Gastroenterology and Metabolism, University of Amsterdam, Amsterdam, Netherlands; ^6^Reference Laboratory for Neonatal Screening, Centre for Health Protection, National Institute for Public Health and the Environment (RIVM), Bilthoven, Netherlands; ^7^Centre for Population Screening, National Institute for Public Health and the Environment (RIVM), Bilthoven, Netherlands; ^8^Department of Clinical Chemistry, Endocrine Laboratory, Amsterdam UMC, Amsterdam Gastroenterology and Metabolism, Vrije Universiteit Amsterdam, Amsterdam, Netherlands; ^9^Department for Vaccine Supply and Prevention Programmes, National Institute for Public Health and the Environment (RIVM), Bilthoven, Netherlands; ^10^Department of Pediatric Endocrinology, Amsterdam UMC, Emma Children's Hospital, University of Amsterdam, Amsterdam, Netherlands; ^11^Department of Pediatric Neurology, Amsterdam UMC, Amsterdam Leukodystrophy Center, Emma Children's Hospital, Amsterdam Neuroscience, University of Amsterdam, Amsterdam, Netherlands

**Keywords:** adrenoleukodystrophy, peroxisomes, newborn screening, neonatal, gender, heel prick, dried bloodspots, X chromosome

In the original article, there was a mistake in the legend for **Figure 6** as published. Three numbers were not in superscript which changed concentrations and there was an error in dosage. The correct legend appears below.

The authors apologize for this error and state that this does not change the scientific conclusions of the article in any way. The original article has been updated.

**Figure 6** | Our patient- and parent-friendly, multidisciplinary, centralized follow-up protocol. ^1^All follow-up appointments will be scheduled on the same day (Wednesday); ^2^Before 10:00 AM; ^3^15 μg/kg/dose, max. 125 μg/dose; ^4^10 μg/dL = 276 nmol/L, 18 μg/dL = 497 nmol/L; ^5^10 mg/m^2^/day in three equal doses (3 times per day 33.3% of the total daily dose); when older than 6 months: 50% early in the morning, and 25% early in the afternoon and evening. Adrenal surveillance protocol adapted and modified from Regelmann et al. (2018). Abbreviations: ACTH, adrenocorticotropic hormone; K, potassium; Na, sodium; PRA, plasma renin activity; Q&A, questions & answers.

## References

[B1] Regelmann M. O. Kamboj M. K. Miller B. S. Nakamoto J. M. Sarafoglou K. Shah S.. (2018). Adrenoleukodystrophy: guidance for adrenal surveillance in males identified by newborn screen. J. Clin. Endocrinol. Metab. 103, 4324–4331. 10.1210/jc.2018-00920 30289543

